# GLP-I secretion in healthy and diabetic Wistar rats in response to aqueous extract of *Momordica charantia*

**DOI:** 10.1186/s12906-018-2227-4

**Published:** 2018-05-18

**Authors:** Gulzar Ahmad Bhat, Haseeb A. Khan, Abdullah S. Alhomida, Poonam Sharma, Rambir Singh, Bilal Ahmad Paray

**Affiliations:** 1 Department of Zoology, HNB Central University Garhwal, Srinagar, Uttarakhand 249161 India; 2 Department of Biochemistry, College of Science, King Saud University, Riyadh, 11451 Saudi Arabia; 3 Department of Zoology, Indira Gandhi National Tribal University, (A Central University), Amarkantak, M.P 484887 India; 4 Department of Biological Sciences, Bundelkhand University, Jhansi, UP India; 5 Zoology Department, College of Science, King Saud University, PO Box 2455, Riyadh, 11451 Saudi Arabia

**Keywords:** Diabetes mellitus, Glucagon-like peptide 1, Wistar rats, *Momordica charantia*

## Abstract

**Background:**

Diabetes mellitus is one of the major global health disorders increasing at an alarming rate in both developed and developing countries. The objective of this study was to assess the effect of aqueous extract of *Momordica charantia* (AEMC) on fasting blood glucose (FBG), tissue glycogen, glycosylated haemoglobin, plasma concentrations of insulin and GLP-1 hormone (glucagon-like peptide 1) in healthy and diabetic wistar rats.

**Methods:**

Male Wistar rats (both normal and diabetic) were treated with AEMC by gavaging (300 mg/kg body wt/day for 28 days).

**Results:**

AEMC was found to increase tissue glycogen, serum insulin and GLP-1 non-significantly (*P* > 0.05) in normal, significantly (*P* < 0.01) in diabetic Wistar rats, whereas decrease in FBG and Glycosylated haemoglobin non-significantly (*P* > 0.05) in normal, significantly (*P* < 0.01) in diabetic Wistar rats. The elevation of GLP-1 level in normal and diabetic treated groups may be due to the L-cell regeneration and proliferation by binding with L-cell receptors and makes a conformational change, resulting in the activation of a series of signal transducers. The polar molecules of *M. charantia* also depolarize the L-cell through elevation of intracellular Ca^2+^ concentration and which in turn releases GLP-1. GLP-1 in turn elevates beta-cell proliferation and insulin secretion.

**Conclusion:**

The findings tend to provide a possible explanation for the hypoglycemic action of *M. charantia* fruit extracts as alternative nutritional therapy in the management and treatment of diabetes.

## Background

Diabetes Mellitus (DM) is one of the most prevalent and serious metabolic disorders affecting about 200 million people worldwide [1]. Type 2 DM (T2DM) is the prevalent form of DM, which poses huge economic burden to all nations, especially the developing countries, which are accounting for more than 80% of reported cases of T2DM [2]. New generation antidiabetic drugs chiefly, α-glucosidase inhibitors, insulin sensitizers, GLP-1 agonists and DPP-4 inhibitors have been developed for effective control and management of T2DM [3].

Glucagon-like peptide (GLP-1) is physiologically important regulators of metabolic control and is an important hormone that stimulates the secretion of glucose-dependent insulin from pancreatic 훽-cells. This endocrine hormone of 30-amino acids is produced in the enteroendocrine L-cells [4]. Is has been reported that the continuous GLP-1 treatment in T2DM improve β-cell function, normalizes blood glucose and restore first-phase insulin secretion and “glucose competence” to β-cells; hence, GLP-1/GLP-1Rs are therapeutic targets for treating T2DM [5]. Recently GLP-1 analogs such as Exenatide, Liraglutide, Metformin etc. have been introduced for therapeutic intervention. However, there are many side effects of these drugs like fluid retention, weight gain, headache, upper respiratory tract infection and urinary tract infection.

The side effects and costly treatment associated with current day antidiabetic drugs, necessitated search for alternate remedy for the treatment of DM. Since ancient times, medicinal plants and their extracts have been effectively used as first line therapy to combat diabetes but are yet to be commercially formulated as modern medicines [6]. Traditional alternative medicine involves the use of herbs and their products as alternatives to chemical drugs.

*Momordica charantia* (MC) commonly known as bitter melon belongs to the family *Cucurbitaceae* is a tropical herbaceous vegatable used for management of DM throughout the world [7, 8]. The antidiabetic effects of MC have been extensively reviewed [9–11]. The possible mechanisms reported include protection of islet 훽-cells [12], increasing insulin secretion [13], inhibition of intestinal 훼-glucosidase [14], and glucose transport [15], activation of AMPK [16, 17], improving insulin resistance [18, 19] and increasing hepatic glucose disposal and decreasing gluconeogenesis [20, 21]. Charantin substance extracted from MC showed hypoglycaemic effect on normal and diabetic rabbits [22]. Bitter melon has been found to increase insulin sensitivity [23]. Decrease in blood glucose, cholesterol and triglycerides through a decrement in PKC-β activity was reported in diabetic rats induced by streptozotocin when MC juice was administered at 6 mL/kg [24]. At the dosage of 375 mg/kg, methanolic fruit extract of MC decreased the fasting blood glucose (FBG) after 12 h in alloxan-induced diabetic rats [25].

In this study, we probed the effects of oral administration of aqueous extract of *Momordica charantia* (AEMC) on fasting blood glucose (FBG), tissue glycogen, glycosylated haemoglobin, serum insulin and GLP-1 in diabetic Wistar rats.

## Methods

### Plant material and its extraction

The fruit of *M. charantia* was purchased from local market. The sample were taken to the Herbarium in Department of Botany, Bundelkhand University, Jhansi, where Dr. Rajdeep Kudesia, confirm its identification and a voucher specimen number (BU-MC- 00712) was placed in the Herbarium. The dried fruit was grinded into powder using grinder and extracted with double distilled water (1:3) by stirring whole night using homogenizer. The extract was filtered and then centrifuged (3000 x g, 10 min) for further purification. The supernatant was further lyophilized for complete dryness to obtain Aqueous Extract of *M. charantia*.

### Chemicals

Streptozoticin (STZ) from Sigma Aldrich was used to induce diabetes. All chemicals and solvents used in this study were purchased from different companies like Merck, Himedia, Rankem, Qualikems, Loba, Sigma and other chemical companies of analytical grade.

### Animals

Two to three months old adult male Wistar rats weighing about 200–250 g (average = 235.5 ± 4.27 g) were purchased from animal DRDE, Gawlior (India). Rats were individually housed in stainless steel wire cages in an animal room with a light: dark cycle (12:12) and temperature (22 ± 2 °C). The Animals were fed pellet diet and water ad-libitum. All procedures described in this study were performed in accordance to the guidelines of Institutional Animal Ethical Committee (BU/ Pharma/ IAEC/12/031).

### Induction of diabetes

In overnight fasted animals, diabetes was induced at 7 am by a single intraperitoneal injection of a freshly prepared solution of streptozotocin (50 mg/kg b.w) in a 0.1 M citrate buffer (pH 4.5). On the third day of STZ-injection, the animals with signs of polyuria and polydipsia and with fasting glycemia (> 200 mg/dl) were considered to be diabetic and included in the study [26].

### Treatment schedule

In the experiment, the rats were randomly divided into 4 groups (*n* = 6/group) as per treatment schedule given below.

Group I (*n* = 6) Normal Control

Group II (*n* = 6) Normal treated with AEMC @ 300mg/kg body weight

Group III (*n* = 6) Diabetic control

Group IV (*n* = 6) Diabetic treated with AEMC @ 300mg/kg body weight 

The control group was given 0.9% normal saline, intramuscularly. Single dose of AEMC was given orally on daily basis for 28 days. Acute study of GLP-1 and serum insulin was done from blood serum in single dose administration of AEMC on first day of experiment. The blood samples were obtained from tail vein at the interval of 0, 20, 30, 40, 50, and 60 min, whereas the sub-chronic study was done following same procedure after 28 days’ administration of AEMC. The 0 min reading indicates taking blood for the estimation of GLP-1 and serum insulin without giving the treatment of AEMC. At the end of the experimental period (after 28 days of treatment), the rats were anaesthetized by chloroform inhalation followed by cervical dislocation and sacrificed as per the guidelines of Institutional Animal Ethics Committee (IAEC). A midline incision was performed at the thoracic region on 29th day of treatment. Trunk blood was collected in heparinized tubes and the plasma was obtained by centrifugation at 5000 rpm for 5 min for the determination of biochemical parameters. The liver and muscle tissues were collected from each group of rats and used for estimation of free and fixed Glycogen.

### Fasting Blood Glucose (FBG)

The blood glucose level of animals was estimated on 0, 14th and 28th day by Glucose Oxidase–Peroxidase Enzymatic Method using a digital glucometer (ACCU-CHEK brand active).

### Glycosilated Haemoglobin (HbA1C)

Glycosylated haemoglobin was estimated by Euro diagnostic system kit based on photometric test at 415 nm using ion exchange resin [27–29].

### Tissue glycogen

This estimation indicates the distinction between free and fixed glycogen content in tissues by using the Anthrone reagent. Immediately after the excision of liver and muscle tissues, 1 g of the tissue were digested within 2 mL of 30% boiling potassium hydroxide (KOH) solution and cooled, and following this 3 mL of 95% ethanol was added and heated until the tissues gets dissolved. Mixtures were cooled and centrifuged at 1000 rpm for 5 min, the supernatant was discarded. The residual material was dissolved in 2 mL of distilled water, and then 10 mL of Anthrone reagent was added and immersed in ice bath to prevent excessive healing. The Tubes were incubated at 100 °C for 4 min for color development and immersed in an ice bath. The absorbance was measured at 620 nm using spectrophotometer.

### Serum insulin

The serum insulin concentrations were measured by an enzyme linked immunosorbent assay (ELISA) method using an ultrasensitive rat insulin ELISA kit (Mercodia, Uppsala, Sweden) in a multi plate ELISA reader (Biorad-680, BIORAD Ltd., Japan).

### Glucagon-Like Peptide-1 (GLP-1)

GLP-1were determined by Ray Bio® Rat GLP-1 ELISA kit given by Toft-Neilsen [30] and Meier et al. [31].

### Statistical analysis

All data were presented as the mean ± SEM. The groups were compared by one-way ANOVA analysis. Data were analyzed by using a statistical software package (SPSS for Windows, version 18) using Tukey’s-HSD multiple range post hoc test. Values were considered significantly different at *p* < 0.05.

## Results

### Effect of *M. charantia* on FBG level in normal and diabetic rats

Oral administration of AEMC 300 mg/kg/day b.w. in normal rats showed non-significant (*P* > 0.05) decrease in FBG on 14 day (5.2%) and 28 day (10.46%) as compared to 0 day in group II, whereas, no change was observed in group I and group III. However, FBG levels showed significant (*P* < 0.01) decrease in group IV on 14 day (41.42%) and 28 day (69.57%) as compared to 0 day readings. On comparing group I with group III, significant increase (*P* < 0.01) in FBG was observed in group III on 14 day (176.16%) and 28 day (286%). However non-significant (*P* > 0.05) decrease in FBG was found on 14 day (23.35%), followed by significant (*P* < 0.01) decrease at 28 day (70.23%) in group IV as compared to group I. At the end of treatment, significant reduction was observed on 14 and 28 day in relation to diabetic control (Group III) (Table 1).

**Table 1:** The effect of *Momordica charantia* on FBG level in normal and diabetic rats

No. of days →	0	14	28
Group I	83.83 ± 2.701	82.5 ± 3.757	85.66 ± 2.929
Group II	82.83 ± 0.401	78.5 ± 0.619^α*^	74.16 ± 0.542^α*^
Group III	301.5 ± 9.570	299.16 ± 31.836^γ**^	310.83 ± 11.652^γ**^
Group IV	304.00 ± 9.292	177.93 ± 7.923^γr^	92.50 ± 13.200^γr^

The values represent as Mean ± SEM for 6 rats each. The results are expressed in mg/dl. α = (*P* > 0.05), β = (*P* < 0.05), γ = (*P* < 0.01) represents comparison of 0 day reading of each group with 14 and 28 days. * = (*P* > 0.05), ** = (*P* < 0.05), *** = (*P* < 0.01) represents comparison of Group-III with Group-I. ^p^ = *P* > 0.05), ^q^ = (*P* < 0.05), ^r^ = (*P* < 0.01) compared Group IV with Group III. Group-I Normal Control; Group-II Normal treated (AEMC); Group-III Diabetic control; Group-IV diabetic treated (AEMC)

### Effect of *M. charantia* on liver glycogen, muscle glycogen and glycosylated haemoglobin in normal and diabetic rats

The effect of AEMC on the Liver Glycogen, Muscle Glycogen and Glycosylated Haemoglobin level of normal and diabetic rats is presented in Table 2. After administration of AEMC in normal rats, liver glycogen level was non-significantly (*P* < 0.05) increased (3.97%) in group II, whereas, significant (*P* < 0.01) decrease was observed (65.16%) in group III compared to group I. However, level of liver glycogen increased significantly (*P* < 0.01) in Group IV (171.42%) when compared to group III. Non-significant (*P* < 0.05) increase in muscle glycogen was found (6.37%) in group II. On contrary, significant (*P* < 0.01) decrease in muscle glycogen was reported in group III (58.48%) when compared to group I. However, muscle glycogen in AEMC treated groups increased significantly (*P* < 0.01) in group IV (110.36%) as compared to group III. The level of HbA1C decreased (9.48%) non-significantly (*P* > 0.05) in group II, whereas in diabetic control the HbA1C showed significant (*P* < 0.01) increase in group III (305.33%) as compared to group I. The diabetic rats when treated with AEMC, the HbA1C percentage significantly (*P* < 0.01) decreased in group IV (65.7%) when compared to group III.

**Table 2:** The effect of *Momordica charantia* on Liver Glycogen, Muscle Glycogen and Glycosylated Heamoglobin in normal and diabetic rats

Parameter	Group I	Group II	Group III	Group IV
Liver Glycogen (mg/g)	23.91 ± 1.74	24.86 ± 1.712^*^	8.33 ± 0.741^***^	22.61 ± 1.795^r^
Muscle Glycogen (mg/g)	19.75 ± 1.109	21.01 ± 1.19^*^	8.2 ± 0.958^***^	17.25 ± 0.909^r^
Glycosylated Hemoglobin (%)	5.06 ± 0.100	4.58 ± 0.170^*^	20.51 ± 5.154^***^	7.021 ± 0.187^r^

The values represent as Mean ± SEM for 6 rats each. * = (*P* > 0.05), ** = (*P* < 0.05), *** = (*P* < 0.01) represents comparison of Group-II, III with Group-I. ^p^ = *P* > 0.05), ^q^ = (*P* < 0.05), ^r^ = (*P* < 0.01) compared group IV with Group III. Group-I Normal Control; Group-II Normal treated (AEMC); Group-III Diabetic control; Group-IV diabetic treated (AEMC)

### Effect of single dose of AEMC on serum insulin level in normal and diabetic rats

Despite that the serum insulin levels were not significantly different (*P* > 0.05) at each time points (Table 3), except, 20 min where serum level was significantly higher in all groups, compared to that at 0 min. In group II, non-significant (*P* > 0.05) increase in serum insulin was observed on administration of AEMC at 20 min (6.41%), and tries to compensate towards normal at 60 min (1.41%). However, in group IV significant (*P* < 0.05) increase (85.36%) was observed at 20 min followed by non-significant increase (*P* > 0.05) at 30 min (75%), which again non-significantly falls towards normal at 60 mins (1.62%). Furthermore, significant (*P* < 0.05) change in serum insulin was observed at each time point (20 min 64.0%, 30 min 18.07%, 40 min 15.67%, 50 min 15.38%, and 60 min 0.0%) in group IV when compared to group III.

**Table 3:** The effect of single dose of *Momordica charantia* on Serum insulin level in normal and diabetic Wistar rats

Time in second	0 min	20 min	30 min	40 min	50 min	60 min
Group I	4.10 ± 0.217	4.20 ± 0.206	4.17 ± 0.210	4.16 ± 0.211	4.14 ± 0.211	4.12 ± 0.212
Group II	4.21 ± 0.288	4.48 ± 0.277^α**^	4.36 ± 0.260^α*^	4.30 ± 0.237^α*^	4.25 ± 0.258^α*^	4.22 ± 0.274^α*^
Group III	1.25 ± 0.239	1.39 ± 0.194^α***^	1.36 ± 0.205^α***^	1.34 ± 0.207^α***^	1.30 ± 0.219^α***^	1.25 ± 0.234^α**^
Group IV	1.23 ± 0.226	2.28 ± 0.399^γr^	1.66 ± 0.352^βr^	1.55 ± 0.236^αr^	1.50 ± 0.241^αr^	1.25 ± 0.227^αr^

The values represent as Mean ± SEM for 6 rats each. The results are expressed in ng/mL. α = (*P* > 0.05), β = (*P* < 0.05), γ = (*P* < 0.01) represents comparison of 0 min reading of each group with 20,30,40,50 and 60 min. * = (*P* > 0.05), ** = (*P* < 0.05), *** = (*P* < 0.01) represents comparison of Group-III with Group-I. ^p^ = *P* > 0.05), ^q^ = (*P* < 0.05), ^r^ = (*P* < 0.01) compared Group IV with Group III. Group-I Normal Control; Group-II Normal treated (AEMC); Group-III Diabetic control; Group-IV diabetic treated (AEMC)

### Effect of sub-chronic administration of *M. charantia*on serum insulin level in normal and diabetic rats

On sub-chronic administration of AEMC in normal rats, non-significant (*P* > 0.05) increase in serum insulin was also observed on administration of AEMC at 20 min (23.3 and 85.1%) and falls towards normal at 60 min (0.40 and 8.15%) in comparison to 0 min in group II and group IV respectively. Significant (*P* < 0.01) increase in serum insulin level was observed (20 min 275.5%, 30 min 229.4%, 40 min 202.2%, 50 min 158.4% and 60 min 144%) in group IV as compared to group III (Table 4).

**Table 4:** The effect of sub-chronic administration of *Momordica charantia* on Serum insulin level in normal and diabetic Wistar rats

Time in second	0 min	20 min	30 min	40 min	50 min	60 min
Group I	4.10 ± 0.217	4.20 ± 0.206	4.17 ± 0.210	4.16 ± 0.211	4.14 ± 0.211	4.12 ± 0.212
Group II	4.16 ± 0.301	5.60 ± 0.263^α^*	5.46 ± 0.278^α^*	4.37 ± 0.296^α^*	4.28 ± 0.292^α^*	4.18 ± 0.299^α^*
Group III	1.25 ± 0.239	1.39 ± 0.194^α^***	1.36 ± 0.205^α^***	1.34 ± 0.207^α^***	1.30 ± 0.219^α^***	1.25 ± 0.234^α^**
Group IV	2.82 ± 0.136	5.22 ± 0.273^γr^	4.48 ± 0.310^γr^	4.05 ± 0.310^**βr**^	3.36 ± 0.204^αr^	3.05 ± 0.195^αr^

The values represent as Mean ± SEM for 6 rats each. The results are expressed in ng/mL. α = (*P* > 0.05), β = (*P* < 0.05), γ = (*P* < 0.01) represents comparison of 0 min reading of each group with 20,30,40,50 and 60 min. * = (*P* > 0.05), ** = (*P* < 0.05), *** = (*P* < 0.01) represents comparison of Group-II, III with Group-I. ^p^ = *P* > 0.05), ^q^ = (*P* < 0.05), ^r^ = (*P* < 0.01) compared Group IV with Group III. Group-I Normal Control; Group-II Normal treated (AEMC); Group-III Diabetic control; Group-IV diabetic treated (AEMC)

### Effect of single dose of *M. charantia* on GLP-1 level in normal and diabetic rats

In the present investigation during short term administration of AEMC, GLP-1 level increased significantly (*P* < 0.01) at 20 min (64.6 and 111.46%) and non-significantly (*P* > 0.05) at 60 min (6.61 and 15.77%) in group II and group IV, respectively, when compared to 0 min readings (Table 5). The GLP-1 level showed significant (*P* < 0.01) decrease at 20 min (35.13%), 30 min (39.8%), 40 min (47.79%), 50 min (48.79%) and 60 min (51.4%) in group III as compared with group I. however, significant (*P* < 0.05) increase in GLP-1 was observed (20 min 19.67%, 30 min 19.52%, 40 min 19.41%, 50 min 18.26% and 60 min 14%) in group IV on comparison with group III.

**Table 5:** The effect of single dose of *Momordica charantia* on GLP-1 level in normal and diabetic Wistar rats

Time in second	0 min	20 min	30 min	40 min	50 min	60 min
Group I	12.00 ± 0.288	15.20 ± 0.970	14.81 ± 0.788	13.81 ± 0.484	12.30 ± 0.463	12.08 ± 0.422
Group II	12.15 ± .669	20.00 ± 1.403^β^***	17.28 ± 0.967^α^***	15.23 ± 0.871^α^*	14.75 ± 0.963^α^*	13.65 ± 1.007^α^*
Group III	5.10 ± 0.289	9.86 ± 0.603 ^γ^***	8.91 ± 0.534 ^γ^***	7.21 ± 0.391 ^β^***	6.35 ± 0.396^α^***	5.82 ± 0.311^α^***
Group IV	5.58 ± 0.733	11.80 ± 1.562^γp^	10.65 ± 1.327^γp^	8.61 ± 1.069^αp^	7.51 ± 0.938^αp^	6.46 ± 0.820^αp^

The values represent as Mean ± SEM for 6 rats each. The results are expressed in pg/mL. α = (*P* > 0.05), β = (*P* < 0.05), γ = (*P* < 0.01) represents comparison of 0 min reading of each group with 20,30,40,50 and 60 min. * = (*P* > 0.05), ** = (*P* < 0.05), *** = (*P* < 0.01) represents comparison of Group-III with Group-I. ^p^ = *P* > 0.05), ^q^ = (*P* < 0.05), ^r^ = (*P* < 0.01) compared Group IV with Group III. Group-I Normal Control; Group-II Normal treated (AEMC); Group-III Diabetic control; Group-IV diabetic treated (AEMC)

### Effect of long term exposure of *M. charantia* on GLP-1 level in normal and diabetic rats

Significant (*P* < 0.01) increase in GLP-1 in sub-chronic experiment was observed at 20 min (51.82%) and tends to be normalize at 60 min (6.61%) in comparison to 0 min in group II. However significant increase in GLP-1 level was depicted at 20 min (306.9%) and then falls towards normal at 60 min (16.5%) in comparison with 0 min in group IV. Long term administration of AEMC in diabetic rats results significant (*P* < 0.05) increase in GLP-1 level from (20mins 306.18%, 30mins 238.18%, 40mins 144.97%, 50mins 62.89%, and 60mins 16.50%) in group V, on comparison with group IV Table 6.

**Table 6:** The effect of long term exposure of *Momordica charantia* on GLP-1 level in normal and diabetic rats

Time in second	0 min	20 min	30 min	40 min	50 min	60 min
Group I	12.00 ± 0.288	15.20 ± 0.970	14.81 ± 0.788	13.81 ± 0.484	12.30 ± 0.463	12.08 ± 0.422
Group II	15.63 ± 0.841	23.73 ± 1.654^γ^***	20.52 ± 1.090^γ^***	18.66 ± 0.667 ^α^***	17.46 ± 0.507 ^α^***	16.33 ± 0.729 ^α^***
Group III	5.10 ± 0.289	9.86 ± 0.603^γ^***	8.91 ± 0.534^γ^***	7.21 ± 0.391^β^***	6.35 ± 0.396 ^α^***	5.82 ± 0.311 ^α^***
Group IV	9.27 ± 0.638	37.72 ± 2.315^γr^	31.35 ± 1.704^γr^	22.71 ± 0.948^γr^	15.10 ± 0.276^βr^	10.80 ± 0.600^αr^

The values represent as Mean ± SEM for 6 rats each. The results are expressed in pg/mL. α = (*P* > 0.05), β = (*P* < 0.05), γ = (*P* < 0.01) represents comparison of 0 min reading of each group with 20,30,40,50 and 60 min. * = (*P* > 0.05), ** = (*P* < 0.05), *** = (*P* < 0.01) represents comparison of Group-III with Group-I. ^p^ = *P* > 0.05), ^q^ = (*P* < 0.05), ^r^ = (*P* < 0.01) compared Group IV with Group III. Group-I Normal Control; Group-II Normal treated (AEMC); Group-III Diabetic control; Group-IV diabetic treated (AEMC)

## Discussion

Our hypothesis that AEMC might exert an incretin effect was supported by data obtained in this study. Fasting blood glucose levels in the normal control group remained unchanged throughout the experimental work. Administration of AEMC to normal rats showed reduction in blood sugar levels from day 1 to day 28. But in case of diabetic treated groups the blood sugar level reached near to normal control within 28 days of experiment. The antidiabetic effect of AEMC may be due to increased utilization of glucose, decreased absorption of glucose from GI tract, control on the insulin secretion or inhibition of the α-glucosidase activity.

AEMC has been reported to inhibit absorption of glucose by inhibiting α-glucosidase and suppressing the activity of disaccharidases in the intestine [32]. Whereas Lal et al. [33] experiment on diabetic rats, reported the hypoglycemic activity of AEMC. Results of other scientists [22, 34, 35] on AEMC treatment are also in conformity with the present work.

The Glycosylated hemoglobin (HbA1c) assay has become the most commonly used measure of chronic glycaemia in epidemiological studies, clinical trials and the management of diabetes. In the present study, the diabetic rats have shown significant increase in HbA1c level as compared to normal control which indicates that diabetic rats have poor glycemic control. The long term administration of aqueous extracts AEMC in diabetic rats results a significant decrease in the HbA1c levels in the diabetic rats, but no significant difference in HbA1c percentage found in normal treated groups. HbA1c levels which were noted in consistent with other reports may be due to low plasma level of insulin or high glucose utilization in the peripheral tissues as reported in the present work. The various experimental reports obtained from recent papers on AEMC [22] and in *Elaeodendron glaucum* [36] concords with our results.

It is well known that in diabetes mellitus there will be marked depletion in glycogen storage in hepatic cells and muscle cells in diabetic rats. Liver glycogen and muscle glycogen is drastically reduced in diabetic group and on administration of AEMC for 28 days in the normal and diabetic rats corrects the glycogen level, but not equivalent to normal control group. The decrease in tissue glycogen may be due to enhanced catabolic process such as glycogenolysis, lipolysis and proteolysis, which are the outcomes of lack of insulin or oxidative stress by diabetes may inactive the oxygen synthase or decrease in GLUT4 transporter protein of muscles and cellular glucose in liver cells. Previous studies have validated that a number of plant materials induced an antidiabetic activity partly through stimulation of hepatic glycogenesis [37, 38]. Thus, the significantly higher liver glycogen content recorded in the Diabetic treated (Group IV) compared to the diabetic control (Group III) specified that AEMC was mediated by stimulating insulin secretion, retarding carbohydrates digestion, or by increasing hepatic glycogen synthesis.

In present investigation diabetic group showed low insulin level than control group which indicates the β-cell failure in diabetic rats. The insulin level increased non-significantly 20 to 60 min in short term administration of AEMC, but in diabetic AEMC treated serum insulin increased significantly. The possible mechanism behind the increase in insulin secretion in acute administration of AEMC might have increased the activity of β-cell receptors or showed insulin like activity, or decreases blood glucose concentrations by acting on GLUT-4. In sub-chronic administration of AEMC, the serum insulin level in diabetic treated groups increased as compared to diabetic group. Serum insulin was also increased in the normal AEMC treated rats. These results are in agreement with the findings of Rotshteyn and Zito [39] and Fernandes et al. [22]. This significant change in serum insulin level in long term exposure of AEMC might be due to increasing the number of β-cell receptors or β-cell proliferation. These results also suggest that AEMC increases the renewal and number of β cells in the pancreas as compared to untreated diabetic rats or may permit the recovery of STZ destroyed β cells and stimulates pancreatic insulin secretion [40]. In support with the present study the elevation of insulin levels was also observed with the administration of AEMC by other authors [36, 41].

Increase in GLP-1 level, from 0 to 60 min was observed in normal and diabetic rats in both short term and long term treatment with AEMC. In diabetic rats highest increase in GLP-1 level was at 20 min of AEMC administration, as compared to 0 min. The results also suggest that induction of GLP-1 on administration of AEMC was more pronounced in diabetic rats as compared to normal. It is possible that in normal rats high increase in GLP-1 is prohibited due to normal physiology and normal level of glucose regulating hormones including GLP-1. Since there is decrease in insulin as well as GLP-1 in diabetic rats, GLP-1 induction is more pounced to decrease enhanced glucose level. These findings indicate that the GLP-1 level is highly significant in sub-chronic administration of AEMC compared to acute administration of AEMC. The Mechanism of elevation of GLP-1 on sub-chronic administration of AEMC in normal and diabetic rats may be due to increased L-cell receptors or L-cell regeneration. The secretion of GLP-1 by AEMC may be due to the presence of sugars, amino acids, small peptides, water soluble alkaloids and plant secondary metabolites, which are known to stimulate GLP-1 secretion [42]. These nutrient molecules activate the enteroendocrine cells of gut by G-protein coupled receptors [43].

In support with the present study Cicero and Tartagni [44] reported that administration of *Beriberis* plant at the dose of 500 mg/kg bw, showed that Berberine is an alkaloid present in *Beriberis* plant that affects glucose metabolism, increasing insulin secretion, stimulating glycolysis, Berberine also increases glucose transporter-4 (GLUT-4) and GLP-1 levels. Other results in support of increase in GLP-1 were observed in *Smallanthus sonchifolius* [45], *Agave tequilana* Gto. and *Dasylirion spp.* [46], *Ilex paraguariensis* [47], *Cinnamomum zeylanicum* [48], *Pinus koraiensis* [49], *Glycine max* [50] and *Berberis* [45].

## Conclusion

Results of present study supported the traditional use of aqueous extract of *M. charantia* significantly for the hypoglycemic action in the management of diabetes.

## Abbreviations

AEMC: Aqueous extract of *Momordica charantia*
AMPK: Adenosine monophosphate activated protein kinase
ANOVA: Analysis of variance
DM: Diabetes mellitus
DPP: Dipeptidyl peptidase
DRDE: Defence research & development establishment
ELISA: Enzyme linked immunosorbent assay
FBG: Fasting blood glucose
GI: Gastrointestinal
GLP-1: Glucagon like peptide
GLUT: Glucose transporter
HbA1C: Glycosilated haemoglobin
IAEC: Institutional animal ethics committee
KOH: Potassium hydroxide
PKC: Protein kinase-C
STZ: Streptozoticin
T2DM: Type 2 diabetes mellitus
