# Correction to: GLP-I secretion in healthy and diabetic Wistar rats in response to aqueous extract of *Momordica charantia*

**DOI:** 10.1186/s12906-018-2252-3

**Published:** 2018-06-12

**Authors:** Gulzar Ahmad Bhat, Haseeb A. Khan, Abdullah S. Alhomida, Poonam Sharma, Rambir Singh, Bilal Ahmad Paray

**Affiliations:** 1 Department of Zoology, HNB Central University Garhwal, Srinagar, Uttarakhand 249161 India; 2 Department of Biochemistry, College of Science, King Saud University, Riyadh, 11451 Saudi Arabia; 3 Department of Zoology, Indira Gandhi National Tribal University, (A Central University), Amarkantak, M.P 484887 India; 4 Department of Biomedical Sciences, Bundelkhand University, Jhansi, UP India; 5 Zoology Department, College of Science, King Saud University, Box 2455, Riyadh, PO 11451 Saudi Arabia

## Correction

Following publication of the original article [1], the authors reported that there was an error in the acknowledgements. In this Correction, the incorrect and correct acknowledgements are shown.

Originally the acknowledgement has been published as:

We are thankful to Department of zoology, Hemvati Nandan Bahuguna Central University Garhwal Uttarakhand, India, for providing necessary facilities.

The correct acknowledgement is:

We are thankful to Department of Zoology, Hemvati Nandan Bahuguna Central University, Garhwal, Uttarakhand, India, and Innovation Centre, Bundelkhand University, Jhansi, India for providing necessary facilities.
